# The epidemiology of paediatric *Mycoplasma pneumoniae* pneumonia in North China: 2006 to 2016

**DOI:** 10.1017/S0950268819000839

**Published:** 2019-05-09

**Authors:** Li-Wei Gao, Ju Yin, Ying-hui Hu, Xiu-yun Liu, Xue-li Feng, Jian-Xin He, Jun Liu, Yan Guo, Bao-Ping Xu, Kun-Ling Shen

**Affiliations:** China National Clinical Research Center for Respiratory Diseases, Department of Respiratory Medicine, Beijing Children's Hospital, Capital Medical University, National Center for Children's Health, Beijing 100045, China

**Keywords:** Epidemiology, Mycoplasma, paediatrics, pneumonia

## Abstract

Paediatric *Mycoplasma pneumoniae* pneumonia (MPP) is a major cause of community-acquired pneumonia in China. Data on epidemiology of paediatric MPP from China are little known. This study retrospectively collected data from June 2006 to June 2016 in Beijing Children's Hospital, Capital Medical University of North China and aims to explore the epidemiological features of paediatric MPP and severe MPP (SMPP) in North China during the past 10 years. A total of 27 498 paediatric patients with pneumonia were enrolled. Among them, 37.5% of paediatric patients had MPP. In this area, an epidemic took place every 2–3 years at the peak, and the positive rate of MPP increased during these peak years over time. The peak age of MPP was between the ages of 6 and 10 years, accounting for 75.2%, significantly more compared with other age groups (*χ*^2^ = 1384.1, *P* < 0.0001). The epidemics peaked in September, October and November (*χ*^2^ = 904.9, *P* < 0.0001). Additionally, 13.0% of MPP paediatric patients were SMPP, but over time, the rate of SMPP increased, reaching 42.6% in 2016. The mean age of paediatric patients with SMPP (6.7 ± 3.0 years old) was younger than that of patients with non-SMPP (7.4 ± 3.2 years old) (*t* = 3.60, *P* = 0.0001). The prevalence of MPP and SMPP is common in China, especially in children from 6 to 10 years old. Paediatric patients with SMPP tend to be younger than those with non-SMPP. MPP outbreaks occur every 2–3 years in North China. September, October and November are the peak months, unlike in South China. Understanding the epidemiological characteristics of paediatric MPP can contribute to timely treatment and diagnosis, and may improve the prognosis of children with SMPP.

## Introduction

Community-acquired pneumonia (CAP) is a major life-threatening disease. *Mycoplasma pneumoniae* infection is one of the most common causes, accounting for 10%–40% [1, 2]. Unfortunately, *M. pneumoniae* pneumonia (MPP) has also increased in China recently. One study showed that *M. pneumoniae* infections made up approximately 70% of all cases of CAP in children over 5 years old [3]. However, there have been no studies with large numbers of paediatric patients with MPP to explore the epidemiology and dynamic changes in the past few years in China. Additionally, most paediatric cases of MPP are benign, but some might develop pleural effusion, multi-organ damage, or serious long-term sequelae, including bronchiolitis obliterans, bronchiectasis or atelectasis [4]. However, the prevalence of severe MPP (SMPP) in Chinese children is also unclear. Therefore, to better understand the epidemiologic features of MPP in paediatric patients in China, we collected data from a large number of hospitalised patients with MPP in the past 10 years to analyse the distributions of gender, age, seasons and other factors.

## Methods

### Study subjects

Paediatric patients aged from 1 month to 18 years with pneumonia as the first diagnosis were retrospectively reviewed from June 2006 to June 2016 in Beijing Children's Hospital, Capital Medical University. They were all Chinese living in North China, including North, Northeast and Northwest China. Demographic features of the patients, clinical information and laboratory data were retrospectively collected from the records of all children. All of the paediatric patients were assigned into five age groups: <1 year, 1–3 years, 3–6 years, 6–10 years and 10 years or older. Four distinct seasons were defined as follows: spring (March–May), summer (June–August), autumn (September–November) and winter (December to the following February). This study was reviewed and approved by the Ethics Committee of Beijing Children's Hospital, Capital Medical University (2017-k-35).

### Diagnostic criteria

The diagnosis of pneumonia is defined as follows [5]: clinical manifestations (fever, cough or wheezing), physical examination and chest imaging with infiltrates. Severe pneumonia defined as pneumonia with one of the followings [6]: (1) poor general condition; (2) increased respiratory rate (infant >70/min, older children >50/min); (3) dyspnoea and cyanosis; (4) multilobe involvement or ⩾2/3 lung involvement; (5) extrapulmonary complication; (6) pleural effusion and (7) transcutaneous oxygen saturation in room air ⩽92%. *M. pneumoniae* infection is defined as single titres of serum *M*. *pneumoniae* antibody ⩾1:320, or seroconversion (increased antibody titres ⩾4 fold) [7]. SMPP is defined as severe pneumonia with *M. pneumoniae* infection. Exclusion criteria were as follows: subjects with a common cold, mild upper-respiratory tract manifestation with no evidence of pneumonia on chest radiograph or chronic pulmonary disease that might affect the chest X-ray results or aspiration pneumonia or interstitial lung disease.

### Detection of *M. pneumoniae*

*M. pneumoniae* antibody was detected using the passive agglutination method (Serodia-Myco II, Fujirebio, Japan). The experimental process was strictly in accordance with the instructions. Positive *M. pneumoniae* infection was defined as single titres of serum *M. pneumoniae* antibody ⩾1:320, or seroconversion (increased antibody titres ⩾4 fold).

### Statistical analysis

All data were analysed using SPSS version 19.0 software. Continuous data are presented as the mean ± standard deviation or range. Continuous variables were analysed using Student's *t* test, and categorical variables were analysed using the *χ*^2^ test or Fisher's exact test if any expected value was below five. *P* value <0.05 was considered statistically significant.

## Results

### Epidemiology of paediatric patients with pneumonia

**Fig. 1.**
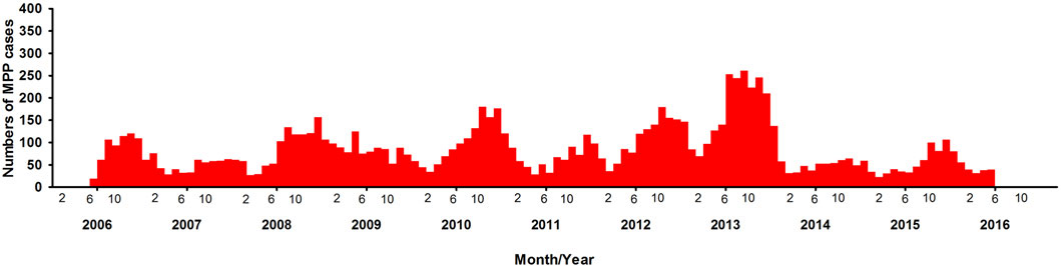
Distribution of children with MPP in different years.

A total of 27 498 children diagnosed with pneumonia were enrolled in this study; among them, 16 346 were male and 11 152 were female, with a male-to-female ratio of 1.5:1. The mean age was 4.1 ± 3.8 years old, and the median age was 3.2 years, ranging from 1 month to 18 years. The percentage of paediatric patients with pneumonia less than 5 years old was about 66.1% (18171/27498). The distribution of children with pneumonia in different years is shown in Table 1 and Figure 1.

**Table 1.** Distribution of children with pneumonia in different years

Radiographic pattern	Cases of pneumonia (*n*)	Cases of MPP (*n*, %)	Cases of SMPP (*n*, %)
June 2006 to May 2007	2119	869 (41.0)	6 (0.7)
June 2007 to May 2008	2014	584 (28.6)	12 (2.1)
June 2008 to May 2009	2974	1299 (43.7)	63 (4.8)
June 2009 to May 2010	2671	796 (29.8)	46 (5.8)
June 2010 to May 2011	2880	1275 (44.2)	189 (14.8)
June 2011 to May 2012	2482	825 (33.2)	127 (15.4)
June 2012 to May 2013	3360	1474 (43.9)	200 (13.5)
June 2013 to May 2014	3658	1882 (51.5)	292 (15.5)
June 2014 to May 2015	2585	553 (21.4)	145 (26.2)
June 2015 to May 2016	2728	743 (27.2)	260 (35.0)
Total	27 498	10 300 (37.5)	1340 (13.0)

### Demographic features of paediatric patients with *M. pneumoniae* pneumonia

A total of 10 300 patients were MPP, nearly accounting for 37.5% (10 300/27 498) of all the patients with pneumonia. There were 5415 boys and 4885 girls, with a male-to-female ratio of 1.1:1. There was a significant difference between boys and girls (*χ*^2^ = 322.5, *P* < 0.0001) (Table 2). During the past 10 years, there have been four outbreaks at the interval of 2–3 years. Over time, the positive rate of MPP has increased in the peak years, reaching to 51.5% from June 2013 to May 2014 (Table 1). The numbers of the patients started to increase at the end of August, with the period of greatest incidence lasting for 5 months every year. This indicated the MPP tended to break out at a particular time, mainly in autumn.

**Table 2.** Age and seasonal distribution of *M. pneumoniae* pneumonia in children

	Pneumonia (*n*)	MPP (*n*, %)	*χ*^2^	*P*
Sex			322.5	<0.0001
Male	16 346	5415 (33.1)		
Female	11 152	4885 (43.8)		
Age group			1384.1	<0.0001
<1 year	8169	98 (1.2)		
1–3 years	4998	709 (14.2)		
3–6 years	5004	2485 (49.7)		
6–10 years	6252	4773 (76.3)		
⩾10 years	3075	2235 (72.7)		
Season			904.9	<0.0001
Spring	6169	1568 (25.4)		
Mar	2117	447 (21.1)		
Apr	2018	474 (23.5)		
May	2034	647 (31.8)		
Summer	5803	2541 (43.8)		
Jun	1815	641 (35.3)		
Jul	1919	863 (45.0)		
Aug	2069	1037 (50.1)		
Autumn	7039	3396 (48.3)		
Sep	2182	1059 (48.5)		
Oct	2300	1163 (50.7)		
Nov	2557	1174 (45.9)		
Winter	8487	2795 (32.9)		
Dec	3243	1211 (37.3)		
Jan	2964	926 (31.2)		
Feb	2280	658 (28.9)		

### Age and season distributions of *M. pneumoniae* pneumonia

**Fig. 2.**
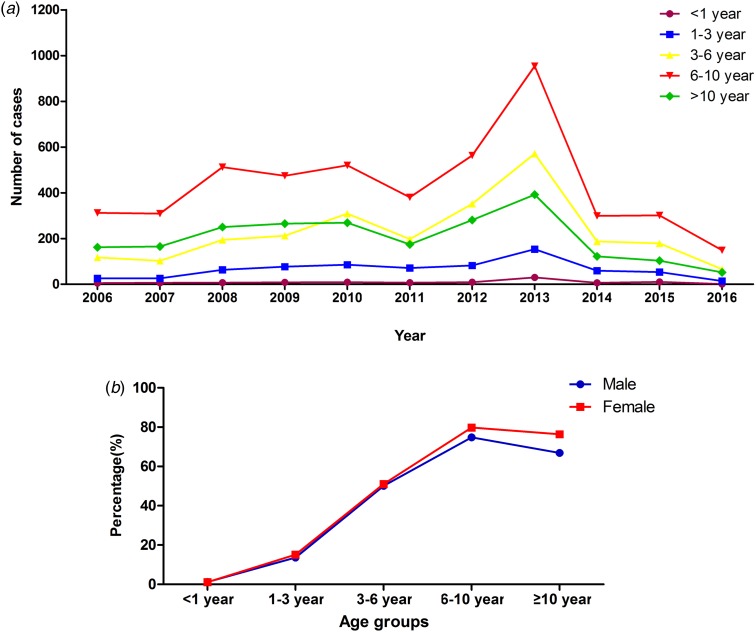
Distribution of MPP patients with different age in different years and gender.

The median age of patients with MPP was 7.4 years (1 month to 18 years). The age group of 6–10 years had the higher rate of MPP than the other age groups (*χ*^2^ = 13 844.14, *P* < 0.0001), accounting for 76.3% of cases of pneumonia. The peak age did not change during the past 10 years (Fig. 2). However, the rate of MPP in peak year increased with time. Autumn had a higher rate of MPP than the other seasons (*χ*^2^ = 904.9, *P* < 0.0001) (Table 2).

### Comparison of severe *M. pneumoniae* pneumonia with non-severe *M. pneumoniae* pneumonia

**Fig. 3.**
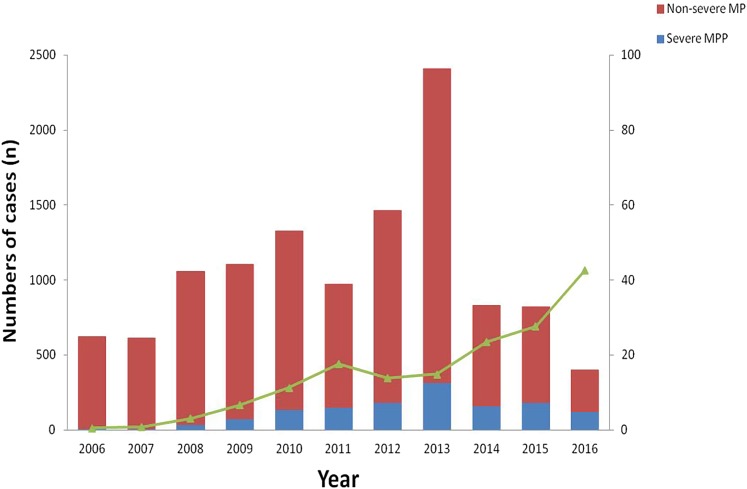
Prevalence of SMPP patients in different years.

Approximately 13.0% (1340/10 300) of patients with MPP were SMPP, and the rate of SMPP was increasing year by year, nearly reaching 42.6% in 2016 (Fig. 3). The mean age of paediatric patients with SMPP (6.7 ± 3.0 years old) was younger than that of patients with non-SMPP (7.4 ± 3.2 years old) (*t*^2^ = 3.60, *P* = 0.0001). The rates of SMPP in patients with MPP in the five age groups (<1 year, 1–3 years, 3–6 years, 6–10 years and more than or equal to 10 years) were 11.2% (11/98), 12.0% (85/709), 15.1% (375/2485), 13.2% (629/4773) and 10.7% (240/2235), respectively. The rate of SMPP was higher in the age group of 3–6 years than the other age groups (*χ*^2^ = 20.74, *P* = 0.0004). Comparison of the age distribution of patients with MPP showed the peak age of paediatric patients with SMPP to be lower than that of patients with non-SMPP (the peak age was 6 to 10 years old). Autumn was the peak season in the SMPP group, as in the non-SMPP group.

## Discussion

*M. pneumoniae* infection is one of the major pathogens in children with CAP. A study performed in the Suzhou Province in South China reported that the rate of *M. pneumoniae* infection was ranging from 30.27% to 36.08% [8, 9]. Our study of north China showed the rate of MPP reached 37.5% in paediatric patients with pneumonia. In addition, we found during the past 10 years, the rate of MPP in the peak years increased, even reaching 51.5% from June 2013 to May 2014. Two to three years will be a peak endemic of MPP in a hospital-based population in North China. Previous studies also showed the same epidemic patterns of MPP in other countries [10]. Eun *et al*. reported the cyclic occurrence of MPP in Korea, with six epidemic peaks in 18 years separated by 3–4 years [11].

The effect of gender on *M. pneumoniae* infection is different. In our study, the positive rate of MPP was higher in girls than in boys in North China, as in South China [12]. That may be because girls are more susceptible to *M. pneumoniae* infection than boys. However, some studies have found that gender is not an important factor for *M. pneumoniae* infection [13]. MPP could occur at any age group, especially in pre-school-aged and school-aged children [14]. Defilippi *et al*. showed the positive rate of *M. pneumoniae* infection in school-aged children to be 41.76% [15]. In Turkey and Japan, the peak age was 7–10 years old [16, 17]. In Australia, the age group most commonly affected by *M. pneumoniae* was those of 5–9 years old [18]. A study of China demonstrated that children over 7 years old had the highest rate in South China [12]. Most studies report the most common age group of MPP to be over 5 years old, as in our study (6–10 years old). This may be related to human immune response after MP infection. We found the peak age did not change in the past 10 years in North China. However, some studies have reported that most patients with *M. pneumoniae* infection are younger than 5 years [19].

Paediatric cases of MPP are found year-round, but different studies have reported different results with respect to the seasons [20]. In our study, we found autumn to be the peak season in North China, while a study in South China showed the MPP rate to be higher in the summer than in the autumn, spring or winter. A Korean study showed the epidemic peaks of MPP to take place in autumn or winter [11]. One study performed in both children and adults in Zagreb showed MPP to be more common between August and November [21]. Two other studies performed in Italy and Australia showed *M. pneumoniae* infection to be more common in June or July [15, 18]. Foy *et al*. found that the endemics of *M. pneumoniae* infection in Seattle have no significant seasonal fluctuations [22]. Another study conducted between 1986 and 2004 in Korea showed that the peak season changed over time [11]. Before 1996, the peak epidemics of *M. pneumoniae* infection took place in summer, while the later epidemics took place in autumn or early winter.

In recent years in China, more and more cases of SMPP have been reported [23]. However, no large case studies have been performed to investigate the prevalence of SMPP. Our study showed that SMPP made up about 13.0%, of all cases, with that proportion increasing over time. The increased positive rate of SMPP in China might be related to the high rate of macrolide-resistance of *M. pneumoniae* (more than 90%) [24, 25]. There was no difference in seasons between SMPP and non-SMPP paediatric patients. The mean age of paediatric patients with SMPP was younger than that of patients with non-SMPP in our study. This suggested that younger paediatric patients are susceptible to SMPP. Therefore, in clinical settings, paediatricians should pay more attention to younger patients with MPP.

## Conclusions

MPP and SMPP are more and more common in North China, especially in children from 6 to 10 years old. Paediatric patients with SMPP tend to be younger than those with non-SMPP. MPP outbreaks occur every 2–3 years in North China. Autumn is the peak season, unlike in South China. Understanding the epidemiological characteristics of paediatric MPP can contribute to timely treatment and diagnosis, and may improve the prognosis of children with SMPP.

## Data

The datasets collected and analysed during the current study are available from the corresponding author upon reasonable request.
